# Towards phasing using high X-ray intensity

**DOI:** 10.1107/S2052252515014049

**Published:** 2015-09-30

**Authors:** Lorenzo Galli, Sang-Kil Son, Thomas R. M. Barends, Thomas A. White, Anton Barty, Sabine Botha, Sébastien Boutet, Carl Caleman, R. Bruce Doak, Max H. Nanao, Karol Nass, Robert L. Shoeman, Nicusor Timneanu, Robin Santra, Ilme Schlichting, Henry N. Chapman

**Affiliations:** a Center for Free-Electron Laser Science, Deutsches Elektronen-Synchrotron DESY, Notkestrasse 85, Hamburg, 22607, Germany; b Department of Physics, University of Hamburg, Luruper Chaussee 149, Hamburg, 22761, Germany; c The Hamburg Centre for Ultrafast Imaging, Luruper Chaussee 149, Hamburg, 22761, Germany; d Biomolecular Mechanisms, MPI for Medical Research, Jahnstrasse 29, Heidelberg, 69120, Germany; e Max Planck Institute for Medical Research, Jahnstrasse 29, Heidelberg, 69120, Germany; f SLAC National Accelerator Laboratory, 2575 Sand Hill Road, Menlo Park, 94025, USA; g Department of Physics and Astronomy, Uppsala University, Box 516, Uppsala, 75120, Sweden; h EMBL, Grenoble Outstation, Rue Jules Horowitz 6, Grenoble, 38042, France; i Laboratory of Molecular Biophysics, Department of Cell and Molecular Biology, Uppsala University, Box 596, Uppsala, 75124, Sweden; j Department of Physics, University of Hamburg, Juniungstrasse 6, Hamburg, 20355, Germany

**Keywords:** serial femtosecond crystallography, high-intensity phasing, radiation damage, electronic damage, X-ray free-electron lasers, high XFEL doses

## Abstract

X-ray free-electron lasers (XFELs) show great promise for macromolecular structure determination from sub-micrometre-sized crystals, using the emerging method of serial femtosecond crystallography. The extreme brightness of the XFEL radiation can multiply ionize most, if not all, atoms in a protein, causing their scattering factors to change during the pulse, with a preferential ‘bleaching’ of heavy atoms. This paper investigates the effects of electronic damage on experimental data collected from a Gd derivative of lysozyme microcrystals at different X-ray intensities, and the degree of ionization of Gd atoms is quantified from phased difference Fourier maps. A pattern sorting scheme is proposed to maximize the ionization contrast and the way in which the local electronic damage can be used for a new experimental phasing method is discussed.

## Abstract 2

Analysis of serial femtosecond crystallography data collected at the Linac Coherent Light Source using two distinct photon fluxes shows different degrees of ionization of Gd atoms bound to a lysozyme protein, due to electronic damage processes. The charge contrast on the heavy atoms is quantified using difference Fourier maps, and the way in which this could be applied to phasing is discussed.

## 1. Introduction   

X-ray free-electron lasers (XFELs) provide extremely bright X-ray pulses of femtosecond duration, that promise to revolutionize structural biology. They can be used to collect diffraction data from sub-micrometre-sized crystals (Chapman *et al.*, 2011 ▸; Boutet *et al.*, 2012 ▸; Redecke *et al.*, 2013 ▸) while outrunning radiation damage with sufficiently short pulses (Neutze *et al.*, 2000 ▸; Kern *et al.*, 2012 ▸; Barty *et al.*, 2012 ▸; Lomb *et al.*, 2011 ▸; Suga *et al.*, 2015 ▸). The room-temperature collection of protein crystallographic data using XFELs is usually performed using the serial femtosecond crystallography (SFX) technique. Given the extremely high intensity of XFEL pulses, each crystal that is hit by an XFEL pulse is completely destroyed. Hence, a new crystal is required for each diffraction pattern. This is typically achieved by injecting the crystals using a stream of liquid, such as a liquid microjet or a thin column of lipidic cubic phase material, into the XFEL interaction region (DePonte *et al.*, 2008 ▸; Sierra *et al.*, 2012 ▸; Weierstall *et al.*, 2012 ▸, 2014 ▸). Each SFX pattern is collected from a different crystal with an unknown orientation and possibly a different size and/or diffraction quality, with the X-ray intensity and spectrum varying pulse to pulse. Moreover, since each XFEL pulse lasts only a few tens of femtoseconds, the crystals are effectively stationary during the exposure, so that only still images are recorded. These fluctuations can be addressed by ‘Monte Carlo integration’, in which a large number of observations are averaged to arrive at accurate structure-factor amplitudes (Kirian *et al.*, 2010 ▸; White *et al.*, 2012 ▸). The resulting amplitudes are accurate enough to allow small features to be resolved such as the difference between amino-acid side chains (Boutet *et al.*, 2012 ▸) or even the anomalous scattering of endogenous sulfur atoms (Barends *et al.*, 2013 ▸). Recently, it was shown that Monte Carlo integrated XFEL data are accurate enough to allow experimental phasing of a protein structure using a heavy-atom derivative for conventional single-wavelength anomalous diffraction (SAD) (Barends *et al.*, 2014 ▸).

The high fluence of XFEL pulses may also enable new phasing approaches (Son, Chapman & Santra, 2011 ▸). Hard X-rays predominantly remove electrons from inner shells, after which relaxation events such as fluorescence and Auger decay fill the resulting core holes. At the high fluences provided by XFEL beams, this relaxation may be followed by a further inner-shell photoionization event, in turn followed by relaxation, resulting in sequences of photoionizations and relaxations so that multiple electrons may be stripped off a single atom, producing very high ionization states (Young *et al.*, 2010 ▸; Rudek *et al.*, 2012 ▸; Murphy *et al.*, 2014 ▸). Depending on sample local structure, the generated photoelectrons may cause a cascade of collisional ionizations within the duration of the pulse, giving rise to over 100 times more valence-shell ionized atoms than the primary inner-shell ionized atoms (Caleman *et al.*, 2009 ▸, 2011 ▸; Ziaja *et al.*, 2015 ▸).

Due to their large interaction cross sections, heavy atoms are predominantly affected by inner-shell photoionization, which changes their scattering properties, whereas the majority of collisional ionizations and the ensuing transfer of energy from the electrons into atomic motions mainly give rise to a decrease in crystalline order and hence an overall decrease in Bragg strength as the dynamics progress (Barty *et al.*, 2012 ▸; Lomb *et al.*, 2011 ▸). This secondary ionization is not particularly sensitive to atomic number, and easily includes every single atom in the sample at doses exceeding about 400 MGy for a protein. In spite of a large amount of electronic rearrangement on heavy atoms at high X-ray intensity, Son, Chapman & Santra (2011 ▸) posited a generalized version of MAD (multiwavelength anomalous dispersion) and suggested that the dose dependence of scattering signals could be used for *de novo* phasing. In addition to the multi-wavelength structure factors, the scheme devised by Son *et al.* requires calculating (or otherwise determining) the atomic scattering factors of the various ionization states of the heavy atoms to allow one to write a set of generalized Karle–Hendrickson equations that can be rigorously solved. Simpler phasing procedures, however, are conceivable, too (Galli *et al.*, 2015 ▸). For example, the bleaching effect of the heavy atoms at high dose reduces their scattering strength, giving the possibility to use this dose-dependent damage for phasing, similar to a radiation damage induced phasing (RIP/RIPAS) scheme in conventional crystallography (Ravelli *et al.*, 2003 ▸; Banumathi *et al.*, 2004 ▸). Alternatively, this bleaching effect can be viewed as a kind of single isomorphous replacement (SIR/SIRAS) (Blow & Rossmann, 1961 ▸; Kartha & Parthasarathy, 1965 ▸). In such a scenario, data collected from a ‘damaged’ structure at a high dose which selectively bleaches heavy atoms are considered the ‘native’ structure, while an ‘undamaged’ data set at a low dose yields the ‘derivative’ structure. In all above phasing approaches, we exploit a large amount of electronic rearrangement on the heavy atoms exclusively occurring during high-intensity XFEL pulses. Therefore we summarize such new phasing approaches under the name of high-intensity phasing (HIP).

Here we investigate the effects of high intensities on heavy atoms contained in protein crystals, and discuss the feasibility of a HIP approach.

## 2. Materials and methods   

### 2.1. Sample preparation and injection   

Rod-shaped microcrystals (≤1 × ≤ 1 × ≤ 2 µm^3^) of chicken egg-white lysozyme (Sigma, Schnelldorf, Germany) were grown as described previously (Boutet *et al.*, 2012 ▸) and stored in a stabilization solution consisting of 8% NaCl in 0.1 *M* sodium acetate buffer pH 4.0. At least 30 min prior to data collection, 100 m*M* gadoteridol [Gd^3+^:10-(2-hydroxypropyl)-1,4,7,10-tetraazacyclododecane-1,4,7-triacetic acid] was added to the crystal suspension. This compound contains a Gd atom, and two gadoteridol complexes can be incorporated per asymmetric unit (Girard *et al.*, 2002 ▸). Before injection, the crystals were left to settle at the bottom of a 15 ml Greiner tube after which the supernatant was removed until the volume of packed crystals was around a third of the total volume. Then, the crystals were resuspended by gentle agitation and injected into the ∼200 nm focus of the Coherent X-ray Imaging (CXI) instrument (Boutet & Williams, 2010 ▸) at the Linac Coherent Light Source (LCLS) using a liquid jet of 4 µm diameter running at 25 µl min^−1^. A rotational anti-settling device (Lomb *et al.*, 2012 ▸) equipped with a thermostat kept the crystal suspension homogeneous at 293 K.

### 2.2. Data collection and processing   

SFX diffraction snapshots were collected in November 2013 (LCLS Run 8, proposal No. LA06) in the nanofocus chamber of CXI at 120 Hz using a Cornell–SLAC Pixel Array Detector (Hart *et al.*, 2012 ▸), which was placed 11.5 cm from the interaction region. Lysozyme microcrystals were hit stochastically by 8.48 keV X-ray pulses of 40 fs duration. Two different data sets were collected over two 12 h shifts: a first ‘low fluence’ (LF) data set was recorded with the X-ray beam attenuated to 1.73% of its full intensity; a second ‘high fluence’ (HF) data set was then collected with the unattenuated SASE beam. To protect the detector from damage due to the high intensities of some of the diffracted beams, a 240 µm-thick flat Si attenuator was placed behind the interaction region. The average XFEL pulse energy during the experiment was 1.6 mJ. Assuming a beamline transmission (intended here as efficiency of the focusing optics) of 30% and a perfect Gaussian spot of 0.2 µm FWHM, the estimated peak X-ray fluence in the interaction region is 7.8 × 10^12^ photons µm^−2^ for the unattenuated beam and 1.3 × 10^11^ photons µm^−2^ for the low-fluence data set, resulting in average doses of 1.27 GGy and 22 MGy, respectively. We note that the photoabsorption cross section for neutral Gd at 8.48 keV is 1.04 × 10^5^ barn, and as such the saturation X-ray fluence for Gd (at which every Gd is photoionized once) is 1/(1.04 × 10^5^ barn) = 9.5 × 10^10^ photons µm^−2^. That is, every Gd atom could be photoionized once on average during the duration of a low-fluence pulse, but high-fluence pulses were up to 82 times higher than the Gd saturation fluence.

The detector geometry was first calibrated using the virtual powder pattern method, followed by a detector geometry refinement (Yefanov *et al.*, 2014 ▸), described in the supporting information. A total of 983 180 crystal diffraction patterns were identified using the *Cheetah* hit finding software (Barty *et al.*, 2014 ▸), with an average hit rate of about 43%. 592 362 of these hits were successfully indexed using the *CrystFEL* software (White *et al.*, 2012 ▸, 2013 ▸). The final Monte Carlo integration resulted in two data sets (see the first two columns of Table 1 ▸ for the statistics of the single sets) that were both truncated to a resolution of 2.1 Å.

### 2.3. Theoretical models   

The X-ray ionization dynamics involving various charge states of heavy atoms induced with a high-fluence X-ray beam can be calculated using the *XATOM* toolkit (Son, Young & Santra, 2011 ▸). Since the HF peak fluence is much higher than the Gd saturation fluence, one may expect that highly charged ions are formed during the X-ray pulse *via* photoionization. Furthermore, every single photoionization event would knock out ∼2–12 electrons from the same atom *via* an Auger cascade. In order to compare with experimental results, we calculated the effective scattering strength of the heavy atom, weighted by the spatial and temporal pulse profile, as
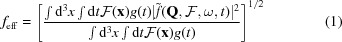
where 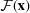 is the X-ray fluence at a given position and 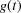 is the temporal pulse shape. 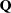 is the photon momentum transfer for a particular scattering direction and 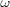 is the photon energy. The dynamical form factor is given by
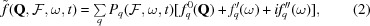
where 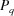 is the time-dependent population of the charge state *q* and 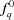 (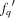 and 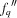) are normal (anomalous) atomic form factors for the ground configuration of the charge state *q*.

Our analysis must take into account the spatial profile of the beam at the interaction region. This profile is assumed to be Gaussian with an FWHM of 0.2 µm on a broad pedestal of much lower fluence but which extends much further (Murphy *et al.*, 2014 ▸). The focused part of the beam is considerably smaller than the average width along the crystals’ shortest side of 1 µm. Even in the case where only half of the incoming photons intersect the crystal, the fluence in that interaction volume may be more than 40 times the saturation fluence for Gd, so that highly charged ions can be created from direct photoionization alone. However, even the low-fluence part of the beam may interact with a large portion of the crystal, contributing to the diffraction signal under lower ionizing conditions. The relative contributions to the total scattered signal from the high and low regions of the beam are given by the ratio of integrated photon counts in those regions (assuming a constant crystal thickness). Although this beam characterization has not been carried out, it was previously found that the ratio of low- and high-fluence regions of the focus at another beamline of the LCLS with similar focusing optics did contain comparable numbers of photons (Murphy *et al.*, 2014 ▸). We also note that high-quality X-ray optics usually exhibit much less than a 50% encircled energy ratio in the core part of the focus. In the absence of a low-fluence pedestal, and considering a flat-top temporal shape (40 fs), the effective scattering strength of Gd in the forward direction is calculated as 57e^−^ for the LF case and 32e^−^ for the HF case. This sets the highest contrast (*i.e.* difference in ionization between the two data sets) achievable to 25e^−^ per Gd. The simulated effective scattering strength does not show strong dependence on the temporal fluctuations of the X-ray pulse, but it is sensitive to its spatial fluence distribution. For example, if the spatial distribution is modelled by a double Gaussian shape (50% hot spot and 50% background with only 0.6 µm FWHM), the effective scattering strength increases to about 59e^−^ for the LF case and 46e^−^ for the HF case, providing a contrast of around 15 electrons.

The cascade of collisional ionizations leads to a much greater ionization of not only Gd atoms, but all atomic species in the sample, and can potentially reduce the contrast of the heavy-atom ionization. The highest-energy photoelectrons are from the light atoms (which have low binding energies). For example, the photoelectron energy from carbon atoms is about 8.2 keV, which can generate almost 400 collisional ionizations within a time of 100 fs (Caleman *et al.*, 2009 ▸, 2011 ▸). The *L*-shell photoelectron energy of Gd is no greater than 1.2 keV (*L* III) which may produce 50 collisional ionizations, but the Gd Auger electrons are of high energy. Although the absorption cross section of C is about 144 times lower than that of Gd, and so the production of photoelectrons per atom is less than for Gd, there are many more C atoms than Gd in the sample. Indeed, this is the case for all the light elements of the sample, and in general the overall generation of the electron cascades scales with the X-ray energy deposited on average per atom, which is proportional to the dose. For the HF dose of 1.27 GGy we expect around 0.5 ionizations per atom on average including electron impact ionization, and around 0.1 ionizations per atom at the LF dose (Chapman *et al.*, 2014 ▸).

The total number of free electrons created increases with time, and is therefore lower with shorter pulses. The effect of Bragg termination, where the diffraction signal is gated as a result of the onset of disorder in the crystal due to random atomic displacement or random ionization, gives rise to a shorter effective pulse duration for the measurement (the later part of the pulse is filtered out of the measurement by selecting just Bragg peaks). We expect that this limits the average ‘ionization background’ experienced at LF and HF, which acts to reduce the contrast of the specific Gd photoionization. We estimate that at HF the Bragg signal is terminated at 20 fs, limiting the average ionization to 0.3, while at LF the Bragg signal will not be terminated during the exposure (Chapman *et al.*, 2014 ▸). If the Gd atoms tend to move slower than the lighter atoms, in a similar fashion to Fe atoms simulated in X-ray-induced explosions of ferredoxin crystals (Hau-Riege & Bennion, 2015 ▸), then these atoms might contribute longer to the Bragg peaks than the disordered structure, with a possible consequence of increasing the effective electron density of Gd at HF. This will further reduce the contrast of Gd density between LF and HF.

## 3. Results   

### 3.1. Analysis of experimental data   

**Figure 1:**
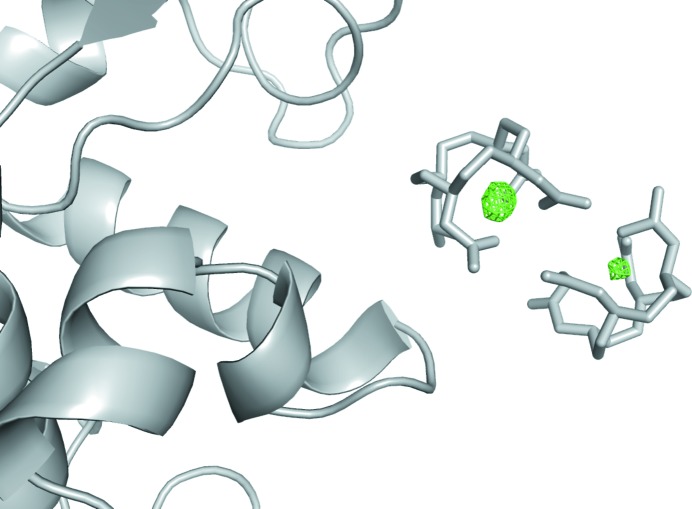
Phased difference (*F*_o_ − *F*_c_) Fourier map, superposed to the lysozyme model deprived of the two Gd ions. Data to 2.1 Å, contoured at 4σ.

The resolution-dependent attenuation of the Si attenuator was corrected in the HF data set after the Monte Carlo integration process by dividing each reflection’s intensity by the calculated transmission factor at the corresponding scattering angle. Structure factors were calculated for the HF and LF data sets using *CCP4 Truncate* (French & Wilson, 1978 ▸) with default options. Cross scaling was performed with *CCP4 Scaleit*, treating the high-fluence data as native and the low-fluence as derivative, since the more heavily ionized Gd atoms, with fewer electrons, can be considered as lighter elements. To visualize the difference in the signal of the Gd atoms, an *F*_o_ − *F*_o_ difference density map was calculated using the lysozyme phases obtained by molecular replacement, performed with *Phaser* (McCoy *et al.*, 2007 ▸). As search model, the structure of Gd-derivatized lysozyme (Protein Data Bank code 1h87, Girard *et al.*, 2002 ▸) was used after the removal of the Gd ions. The map, displayed in Fig. 1 ▸, shows two high peaks at the Gd locations. One peak is higher than the other (9.0σ *versus* 6.2σ), probably due to the higher occupancy of the site (Girard *et al.*, 2002 ▸). In order to estimate the relative number of electrons making up the difference between the two data sets at the Gd positions, two separate molecular-replacement runs were performed, using a search model from which the two Gd ions and part of a tryptophan (Trp) residue (48 electrons in total) had been removed (see the supporting information for details). No significant change in the *B* factors (global and local around the omitted regions) was observed in the two separately refined structures. This finding is important for a quantitative comparison of the electron densities of the omitted parts. *F*_o_ − *F*_c_ maps were calculated around the two missing regions, and these positive difference electron densities were volume integrated. The ratio between the integrated densities around the Gd and the Trp, multiplied by the number of missing electrons at the Trp location, gives an estimate of the effective scattering strength of the two Gd ions. Considering the average occupancy of the two sites, we found that the difference between the two data sets was around 8.8e^−^ per Gd. By repeating the same procedure with other Trp present in the protein, an estimation was made of the error associated with the number of electrons, which is around 20%.

**Figure 2:**
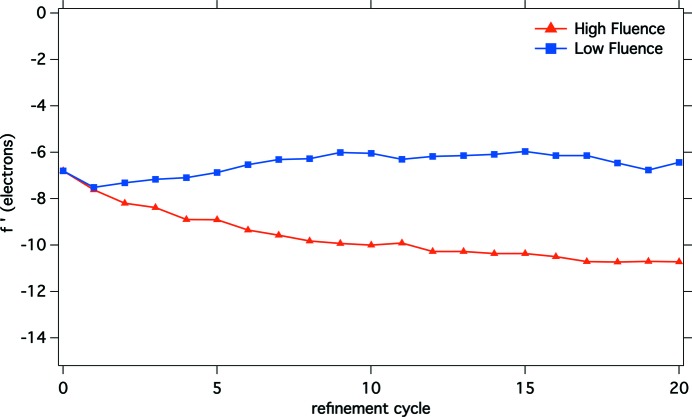
The resulting effective scattering strength of the single Gd ion at the end of each refinement cycle.

Another piece of qualitative evidence of the ionization provoked by the FEL radiation comes from the 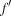 and 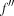 refinement. This was performed with *phenix_refine* (Adams *et al*., 2010 ▸), starting from the anomalous differences (DANO) values and the phases from the best refined model. 20 cycles of alternated real-space and 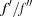 refinement of the two Gd atoms were performed for the LF and HF data. Fig. 2 ▸ displays the resulting scattering strength of the single Gd ion as a function of the refinement cycle, suggesting that the ionization is higher for the HF set, with a difference of about 5 electrons.

**Figure 3:**
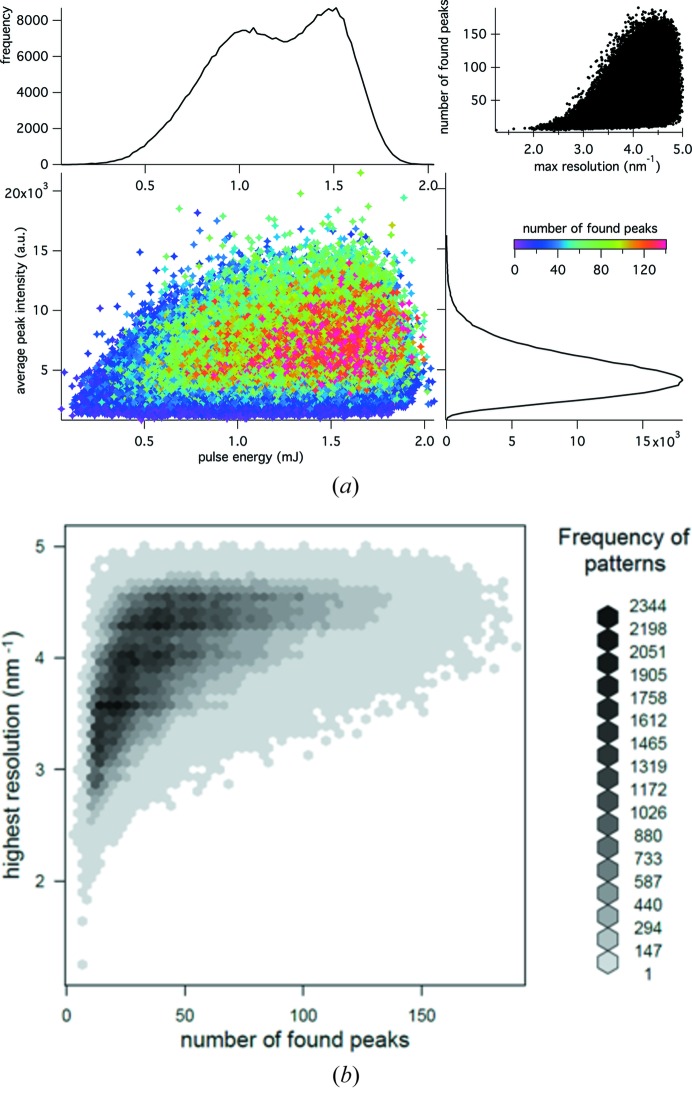
(*a*) Scatter plot of the average intensity of found peaks against the pulse energy, for the high-fluence data set. Each point corresponds to a single indexed diffraction pattern. The colours refer to the number of Bragg peaks found in the pattern (also shown in the upper-right plot, as a function of the maximum resolution found in the corresponding diffraction pattern). The black curves are the projected histograms of the values of the corresponding axis. (*b*) Discrete density plot of the number of found peaks *versus* the highest resolution found. Each hexagonal cell is coloured corresponding to the frequency of patterns in that region.

Due to the stochastic nature of the FEL operation, and the uncertain position, size and shape of the focus, the nominal ‘high-fluence’ data set is aggregated from a mixture of different fluences and therefore a mixture of doses. A similar but less dramatic result applies to the low-fluence data set, since the fluence is not high enough to cause a significant change of the scattering factors. In order to optimize the difference signal in the single-wavelength HIP method, the difference between the X-ray fluences must be the highest possible. To achieve this, we sorted the indexed diffraction snapshots to select only the patterns with the highest fluence. The narrow size distribution of the lysozyme microcrystals means that the observed diffracted intensity should be proportional to the fluence impinging on the crystal (see Lomb *et al.*, 2011 ▸) except for the consideration of the beam’s spatial profile as discussed above. We used the number and average integrated intensity of peaks detected in the patterns, combined with readings from a pulse intensity monitor located upstream of the focusing mirror, to find the snapshots corresponding to the highest dose. These values are represented as a scatter plot in Fig. 3 ▸(*a*), showing a correlation between the number and the average peak intensity on the one hand, and the beam energy on the other. In particular, bright diffraction patterns are mostly found for high-intensity X-ray pulses, and often present a large number of Bragg spots. Furthermore, a high number of Bragg peaks also favours the highest-resolution patterns, as shown in Fig. 3 ▸(*b*), probably selecting also the best diffracting crystals. These brighter diffraction patterns were selected from the HF set as described in the supporting information, from which a new data set of 121 917 patterns labelled ‘HF_best’ was created. This set still presented a satisfactory data quality (see the third column of Table 1 ▸ and the comparison reported in Table 2 ▸). The previous analysis was repeated comparing the new HF_best to the full LF set, showing a higher ionization degree of the Gd atoms, corresponding to 12e^−^, consistent with the difference maps that also showed peaks at slightly higher sigma levels (9.2 and 6.3σ).

### 3.2. Phasing   

Phasing by SAD was performed with *phenix.autosolve* (Adams *et al.*, 2010 ▸) and was accomplished in a straightforward manner for both X-ray fluences. Interestingly, the LF data had a slightly lower *R* factor than the high-fluence data (see the supporting information), even though the latter had a better *R*_split_ metric (Table 2 ▸). This could be an indication of the ionization dynamics effect on the anomalous signal of the heavy atoms (Son *et al.*, 2013 ▸).

The experimental data at different fluences can be considered to a first approximation as a RIP/RIPAS data set, using the HF data as the ‘damaged’ set and the LF as the ‘undamaged’ (Galli *et al.*, 2015 ▸). Several phasing attempts using this strategy were carried out, without success. In the RIPAS approach, the phase information only came from the huge anomalous signal from the heavy atoms, which made SAD phasing straightforward, while any possible isomorphous difference contributed only destructively to the phasing process, making the final result worse than the SAD approach alone. The two ions cannot be located in the RIP (SIR) approach, and even when the correct positions of the Gd ions were given as input no phasing solution was obtained. We believe the main reason is the high discrepancy between the two data sets, as also observed in the large isomorphous *R* factor (see the supporting information). This discrepancy might be caused by an inappropriate scaling procedure, due to Bragg termination (Barty *et al.*, 2012 ▸; Lomb *et al.*, 2011 ▸) which could have the same effect as non-isomorphism. In particular, while Barty *et al.* indicated an isotropic effect that might be compensated by more sophisticated scaling procedures, Lomb *et al.* observed non-scalable changes in individual structure factors.

## 4. Discussion and conclusions   

We have shown that there is a contrast in the effective scattering strengths between low- and high-fluence data recorded with XFEL pulses. The theoretical model of an irradiated isolated Gd atom predicts an effective ionization of between 15 and 25 electrons, whereas the experimental analysis for the Gd derivative of lysozyme shows an average charge state between +8.8 and +12. We suggest the following potential reasons for this large discrepancy: (i) independent-atomic model in theory, (ii) Bragg-peak self-termination, (iii) ionization-induced dynamic fluctuations of the scattering form factors, (iv) unknown X-ray beam intensity profile and (v) ambiguity in the scaling procedure.

Firstly, our theoretical model is based on isolated-atom calculations. Charge rearrangement and local plasma formation that might occur in a molecular environment are not included in our model. Electron transfer from neighbouring atoms to the highly charged heavy atom will affect the effective scattering strength of the heavy ion, as well as the ionization dynamics. Thus a rigorous treatment for the charge rearrangement would be necessary. To estimate the plasma environment effect we performed plasma simulations using *CRETIN* (Scott, 2001 ▸), similar to those described in Caleman *et al.* (2014 ▸). This approach considers the plasma environment, including effects such as continuum lowering and ionization by secondary electrons. The simulations of a system containing Fe as a heavy atom with and without collision ionizations suggested that the secondary collision ionizations would effectively reduce the difference in ionization of heavy atoms between the LF and the HF experiments (see the supporting information for detailed estimation).

Secondly, another effect that could further reduce the ionization contrast is the turning off of the Bragg signal due to the loss of coherent scattering (Barty *et al.*, 2012 ▸). In the HF case we expect that only the first 20 fs of the pulse will actually contribute to the Bragg signal, so relaxation effects taking place after that time range do not contribute to the total ionization. Taking into account only the first 20 fs of the pulse, the previous theoretical model of an isolated Gd atom predicts a difference of the effective scattering strengths as 20 electrons. Combined with the double Gaussian spatial shape of the pulse, this difference becomes around 11 electrons. Additionally, if the disordering of the heavier Gd atoms is less than that of the light atoms and they continue to contribute to Bragg peaks for times beyond 20 fs, then the effective electron density of Gd would be enhanced, diminishing the effect of ionization.

Thirdly, the effect of ionization-induced fluctuations (Son *et al.*, 2013 ▸) is not addressed in standard crystallographic software. During the intense X-ray pulse, the scattering form factors of heavy atoms are stochastically and dramatically changed as a function of time through strong ionization. Therefore, a time-dependent form factor has to be introduced, as shown in equation (2), in order to properly calculate the scattering intensity when an intense X-ray pulse is applied. In conventional X-ray crystallography, however, the time dependence of the form factor has not been taken into account and the deviation due to dynamic fluctuations has been neglected. If we assume the same beam properties as before, the calculated standard deviation of the effective scattering strength for a Gd atom is 5.9e^−^ for the LF case and 10.6e^−^ for the HF case. Without considering these large deviations, the effective scattering strength analysed by the standard crystallographic software would be overestimated (see the supporting information for detailed expressions).

Fourthly, to analyse the scattering signal and electronic damage at high X-ray intensity, it is important to know the X-ray fluence irradiating individual atoms. If some X-ray beam parameters are unknown, proper volume averaging cannot be performed. Our calculations of the effective scattering strength show a strong dependence on the interaction volume geometry, suggesting the need for a calibration of the X-ray beam profile. Another issue is the position dependence of the X-ray fluence across the microcrystal. If the crystal size is larger than the intense spatial profile of the X-ray beam, the heavy atoms in different positions within the same crystal experience a range of fluences. In this case, it may not be possible to define a useful effective form factor for the heavy atoms, as the electronic fluctuations may be expected to be even larger.

Lastly, another problem in treating our data with standard crystallographic software lies in the scaling procedure of SFX data exposed to very high X-ray fluence. This is because the ionization mechanisms of the light atoms, which result in an overall decrease in scattering strength of the molecule, may not be fully corrected for. Similarly, Bragg termination effects may introduce changes in the scattering factors, as mentioned above, and their resolution dependence is not compensated by standard Wilson-type scaling procedures, as shown by Lomb *et al.*

We have shown both experimentally and theoretically that the effective scattering strength of the heavy atoms is dramatically reduced in the high-fluence data set due to ionization dynamics. This reduction can hinder standard experimental phasing approaches, but the contrast between the low- and high-fluence data sets may potentially be used for novel high-intensity phasing methods. In order to accomplish high-intensity phasing, the ionization contrast on the heavy atoms must be maximized. We suggest that the low fluence (for the ‘undamaged’ set) needs to be lower than the one-photon absorption saturation limit, and shorter pulse durations to those used here should avoid the reduction of the ionization contrast by the secondary ionization effects and the self-termination of the Bragg signal. We have shown that by sorting the diffraction patterns according to the impinging X-ray fluence, the ionization contrast can be enhanced. A precise estimation of the beam intensity profile and the adoption of extra diagnostic tools will allow a more effective sorting. Finally, we advise that an experimental determination of new anomalous coefficients at different XFEL intensities is needed to maximize the success of experimental phasing at high fluences.

## 5. Related literature   

For related literature for this paper, see: Emsley *et al.* (2010 ▸), Chen *et al.* (2010 ▸), Immirzi (1966 ▸), McCoy *et al.* (2007 ▸), Langer (2008 ▸), Kabsch (2010 ▸), Zaefferer (2000 ▸).

## Supplementary Material

Supporting information file. DOI: 10.1107/S2052252515014049/gq5004sup1.pdf

## Figures and Tables

**Table 1:** Data collection statistics

	Low fluence (LF)	High fluence (HF)	High fluence strongest diffracting patterns (HF_best)
Space group	*P*4_3_2_1_2		
Unit-cell parameters	*a* = *b* = 79.2(7), *c* = 39.4(4) = = = 90		
Resolution ()	56.01.9	56.02.08†	56.02.08†
Indexed images	218598	373764	121917
Completeness‡ (%)	100 (100)	100 (100)	100 (100)
SFX multiplicity‡	2695 (1346)	4643 (1400)	1512 (466)
*I*/(*I*)‡	18.17 (6.64)	23.60 (8.23)	15.32 (1.59)
*R*_split_ ‡ (%)	8.88 (13.83)	4.92 (12.82)	8.49 (19.46)
CC‡	0.98 (0.97)	0.99 (0.97)	0.98 (0.93)
CC_ano_ ‡	0.64 (0.44)	0.81 (0.47)	0.59 (0.20)
*R*_ano_/*R*_split_ ‡	2.50 (1.61)	3.94 (1.72)	2.35 (1.34)

† Resolution limited by the mask applied (see the supporting information).

‡ Treating Friedel mates as individual measurements.

**Table 2:** *R*_split_ in resolution bins (White *et al.*, 2012 ▸)

1/*d* centre (nm^1^)	Resolution ()	*R*_split_ LF (%)	*R*_split_ HF (%)	*R*_split_ HF_best (%)
1.542	6.48	3.04	2.55	4.23
2.919	3.43	3.78	2.97	4.98
3.487	2.87	4.32	3.25	5.11
3.909	2.56	4.46	3.73	5.93
4.253	2.35	5.36	4.24	6.53
4.549	2.20	6.74	4.85	7.19
4.811	2.08	10.51	12.82	19.46
5.046	1.98	86.13	112.70	127.16
