# Supplementary material for: Towards phasing using high X-ray intensity
Source: IUCrJ. 2015 Sep 30;2(Pt 6):627–34. doi: 10.1107/S2052252515014049 (PMC4645107; doi:10.1107/S2052252515014049)

IUCrJ

Volume 2 (2015)

Supporting information for article:

Towards phasing using high X-ray intensity

Lorenzo Galli, Sang-Kil Son, Thomas R. M. Barends, Thomas A. White, Anton Barty, Sabine Botha, Sébastien Boutet, Carl Caleman, R. Bruce Doak, Max H. Nanao, Karol Nass, Robert L. Shoeman, Nicusor Timneanu, Robin Santra, Ilme Schlichting and Henry N. Chapman

S1. Pre-processing and indexing

Background subtraction and detector correction was performed with the Cheetah software. Pedestal signal arising from the detector was removed by subtracting an average dark image from each frame. Hot pixels were identified and masked, as well the coherent scattering from the liquid jet, which can give rise to strong diffraction at low angles, in the direction perpendicular to the jet. For the high fluence dataset, the edges of the detector were also masked, to cover the possible scattered signal from the edge of the Si attenuator, limiting the highest resolution to about 2.1 Å.

Cheetah was also used to discriminate the patterns containing crystal diffraction, named “hits”, from the rest of the blank shots, by locating pixel regions that lie above a given threshold. Frames containing more than 20 detected peaks were saved as hits. Only these images were processed by the CrystFEL software package (version 0.5.2). This software was utilized for indexing and integrating the Bragg intensities via Monte Carlo methods (Kirian *et al.*, 2010). Indexing was performed with the “mosflm” and “dirax" algorithms, using the peaks location found by the “zaef” gradient search method (Zaefferer, 2000). The unit cell parameters were determined utilizing a subset of the collected data. Subsequent indexing was performed comparing the resulted unit cell parameters to the determined ones, allowing a tolerance of 10% in axis length and 2 degrees in angle.

Bragg intensities were integrated around a radius of 3 pixels centred at the predicted peak location, using an outer annulus between 5 and 6 pixels radius to estimate the background subtraction. The detector geometry and the sample-to-detector distance were further refined at this stage using a special script, by minimizing the distance between observed and predicted peak locations, through translations and rotations of individual detector tiles, as well as by modifying the detector distance to the interaction region. Successive CrystFEL runs were performed followed by finer detector geometry corrections to get the final stream of processed data. As a general trend, the number of indexed frames increased with each iteration of geometry refinement. The complete set of scattered intensities was obtained by merging all the reflections which were integrated at least 10 times, in the point group 422, keeping the Friedel pairs separated. As reported in the tables Table S1, Table S2, Table S3, the $I/\sigma(I)$ values were very high in all resolution shells. The low fluence dataset, which was not limited in resolution by the applied mask at the detector edges, shows that the observed diffraction was limited geometrically by the detector, to a resolution of 1.9 Å.

The reflection lists were first converted to XDS ASCII via the “create-xscale” script distributed with CrystFEL then to the CCP4 file format using XDS “xdsconv” (Kabsch, 2010).

The quality metrics used (Rsplit, Riso) were calculated using CrystFEL "compare_hkl", while the I/sigI was generated by "check_hkl". Isomorphous differences between the LF and HF sets were calculated with Scaleit, and are reported as a function of the resolution in Figure 1S.
The subset of patterns at high fluence ("high fluence best") was selected from the stream of indexed images, by requiring a pulse intensity higher than 1mJ (as recorded from the gas detector), an average peak intensity (expressed as the sum of all integrated peaks intensity divided by the number of them) greater than 4000 detector units, and more than 40 found peaks in the pattern.

S2. Structure determination

The SFX data was phased by molecular replacement using Phaser (McCoy *et al.*, 2007) using the Protein Data Bank ID, 1H87 as a search model, followed by model building in COOT (Emsley *et al.*, 2010) and REFMAC5 (McCoy *et al.*, 2007). The structure was refined at a resolution of 1.9 Å using the data collected at low fluence, to an R-factor of 19.9% (Rfree = 22.2%). The final model was then checked with Molprobity (Chen *et al.*, 2010). The refined structure and the corresponding dataset have been deposited in the Protein Data Bank.

S2.1. Anomalous phasing

SAD phasing was performed with the automatic pipeline of Phenix Autosolve, followed by automatic building cycles in ARP/wARP (Langer, 2008). Low and high fluence data were input as unscaled and unmerged mtz files, and they were solved separately at 2.1 Å resolution. Table S4 lists the scoring for the various phasing steps. SIRAS was conducted in a similar way, with the option to look for anomalous differences. The final structures and the corresponding structure factors have been deposited in the Protein Data Bank.

S3. Estimation of the effective scattering of Gd at HF and LF

The Fo(LF)-Fo(HF) map , generated with FFT (Immirzi, 1966), is presented in figure Figure S2. This shows a localized ionization difference around the Gd ions.

The model lacking of the two Gd ions and the indole group of the Trp residue was generated from the previously refined structure. Two separated restrained refinement runs with REFMAC5 at 2.1 Å, using the structure factors at high and low fluence previously scaled with Scaleit, gave two separated structures with no substantial differences in terms of B factors. Cuboidal volumes of the Fo-Fc maps from these runs, generated with FFT, were selected to cover the gadoliniums or the missing atoms of the Trp, extending the map by 1.5 Å. The maps were integrated using an *ad hoc* script around the volume in which the density was above 1 σ, to minimize the contribution of the noise. The ratio

between the integrated densities was multiplied by the number of electrons of the missing atoms in the Trp and then by the average occupancy of the gadoliniums (respectively of 0.82 and 0.74), calculated by averaging the occupancy resulting from 6 different single-crystal datasets from macrocrystals, crystallized in different conditions and exposed to different radiation sources, in order to mimic the possible anisomorphism of the SFX data (the occupancy refinement was performed with Phenix Refine (Adams *et al.*, 2010)). The resulting number is an estimation of the average “effective” number of scattering electrons in the region of the two heavy atoms. The difference between the results at low fluence and high fluence is the effective ionization of the two sites. This procedure was repeated for 4 different Trp residues (Trp 28, 62, 108 and 111, as labelled in the deposited structure), in order to estimate the error associated with the procedure.

The f’ and f’’ refinement was performed with Phenix Refine, starting from the DANO values and the phases from the best refined model, using 20 cycles of alternated real space and f’/f’’ refinement of the two Gd.

S4. Estimation of the plasma environment effect

To estimate the effect of the surroundings we performed simulations using a non-local thermal equilibrium plasma code – CRETIN (Scott, 2001). The simulations were done similarly to those described in (Caleman & al, 2014). This approach has the advantage that it considers the plasma environment, including effects such as continuum lowering and ionization by secondary electrons. Unfortunately, the atomic model of Gd within CRETIN does not include a sufficiently accurate description of the atomic levels. For a qualitative analysis of how the plasma environment affects the ionization, we considered a system containing Fe instead of Gd. During the x-ray exposure secondary ionization will generate a large number of free electrons, which will increase the ionization of all the atoms in the system. This effectively reduces the difference in ionization between the LF and the HF experiments. To visualize the effect of the secondary collision ionizations from the electrons we have performed plasma simulations with and without collision ionizations. Figure S3 shows that in the absence of collisions, the average ionization of Fe at low fluence is underestimated by a factor of three, while for the high fluence case the ionization is saturated even when the secondary effects are disregarded. We assume that this discussion holds for Gd as well, which would to some extent explain why we observed a smaller difference in ionization in the HF and the LF case compared to estimates based on isolated atoms. By using short x-ray pulses, it may be possible to reduce the ionization effects due to the plasma environment (Son *et al.*, 2011).

S5. Scattering intensity including ionization-induced fluctuation

Assuming that only the heavy atoms scatter anomalously and that they undergo ionization dynamics independently, we can express the scattering intensity with the time-dependent form factor and its fluctuations as (Son *et al.*, 2013):

$$I \propto \int dt\, g(t) \left| F_P{}^{\circ} + \tilde{f}(t) \sum_{j=1}^{N_{\mathrm{Gd}}} e^{i\vec{Q}\cdot\vec{R}_j} \right|^2 + N_{\mathrm{Gd}} V_1, \qquad \text{Eq. (S1)}$$

where $F_P{}^{\circ}$ is molecular form factor for the protein without Gd atoms and N_{Gd} is the number of Gd atoms in a crystal. The fluctuation is given by $V_1 = \int dt\, g(t) \left[\sum_q P_q(t) |f_q|^2 - \left| \sum_q P_q(t) f_q \right|^2 \right]$. The dependences on the momentum $\vec{Q}$, the fluence $\mathcal{F}$, and the photon energy ω are omitted for simplicity. The time-dependent form factor $\tilde{f}(t)$ is dynamically synchronized for all heavy atoms, thus contributing to the coherent signal. Even though the fluctuations from this form factor increase as a function of the fluence, they correspond to a diffuse background. On the other hand, if one introduces a time-averaged form factor, $\bar{f} = \int dt\, g(t) \tilde{f}(t)$, then the scattering intensity is written as:

$$I \propto \left| F_P{}^{\circ} + \bar{f} \sum_{j=1}^{N_{\mathrm{Gd}}} e^{i\vec{Q}\cdot\vec{R}_j} \right|^2 + N_{\mathrm{Gd}} V_1 + \left| \sum_{j=1}^{N_{\mathrm{Gd}}} e^{i\vec{Q}\cdot\vec{R}_j} \right|^2 V_2, \qquad \text{Eq. (S2)}$$

which contains two different types of fluctuations, V_1 and V_2. The latter is the dynamical fluctuation given by $V_2 = \int dt\, g(t) |\tilde{f}(t)|^2 - \left| \int dt\, g(t) \tilde{f}(t) \right|^2$, which contributes to the coherent sum over heavy atoms. If we assume the same beam properties as those used in Sec. 2.3, the calculated standard deviation $\sqrt{V_2}$ for a Gd atom is 5.9e$^-$ for the LF case and 10.6e$^-$ for the HF case. In conventional x-ray crystallography, Eq. (S2) without considering V_1 and V_2 has been used to fit to the scattering intensity measurement. As a result, the effective scattering strength, $|\bar{f}|$ in this case, analysed by the standard crystallographic software would be overestimated because it neglects a large contribution from V_2.

Table S1 Quality of the datasets used : high fluence, HF (373,764 indexed patterns)

1/d centre (nm^{-1})	Resolution (Å)	Completeness	Redundancy	I/σ(I)
1.542	6.48	100	5728.5	40.47
2.919	3.43	100	5105.8	32.70
3.487	2.87	100	5204.2	28.99

3.909	2.56	100	4796.6	25.25
4.253	2.35	100	5018.5	22.28
4.549	2.20	100	4550.7	18.42
4.811	2.08	100	1261.5	7.58
5.046	1.98	49.10	87.9	1.98

Table S2 Quality of the datasets used: high fluence strongest diffracting patterns, HF_best (121,917 indexed patterns)

1/d centre (nm^{-1})	Resolution (Å)	Completeness	Redundancy	I/σ(I)
1.542	6.48	100	1822.3	25.23
2.919	3.43	100	1663.8	20.90
3.487	2.87	100	1702.9	18.78
3.909	2.56	100	1567.8	16.42
4.253	2.35	100	1641.7	14.55
4.549	2.20	100	1479.3	12.06
4.811	2.08	100	403.6	4.93
5.046	1.98	41.09	30.9	1.09

Table S3 Quality of the datasets used : low fluence, LF (218,598 indexed patterns)

1/d centre (nm^{-1})	Resolution (Å)	Completeness	Redundancy	I/σ(I)
1.542	6.48	100	3468.9	30.53

2.919	3.43	100	3016.0	25.52
3.487	2.87	100	3002.9	22.12
3.909	2.56	100	2799.9	19.06
4.253	2.35	100	3055.3	17.06
4.549	2.20	100	2796.2	13.89
4.811	2.08	100	1964.8	9.31
5.046	1.98	100	1253.3	6.13

Table S4 Results from SAD phasing, using the HF (373,764 indexed patterns) and LF (218,598 indexed patterns) datasets.

	high fluence	low fluence
FOM (solve)	0.573	0.553
R factor (solve)	0.3417	0.3160
Score (ARP/wARP)	0.965	0.968
R factor	0.220	0.204
R free	0.280	0.271

Figure S1 Isomorphous differences between the HF and LF datasets expressed with the R factor and weighted R factor (Wted).

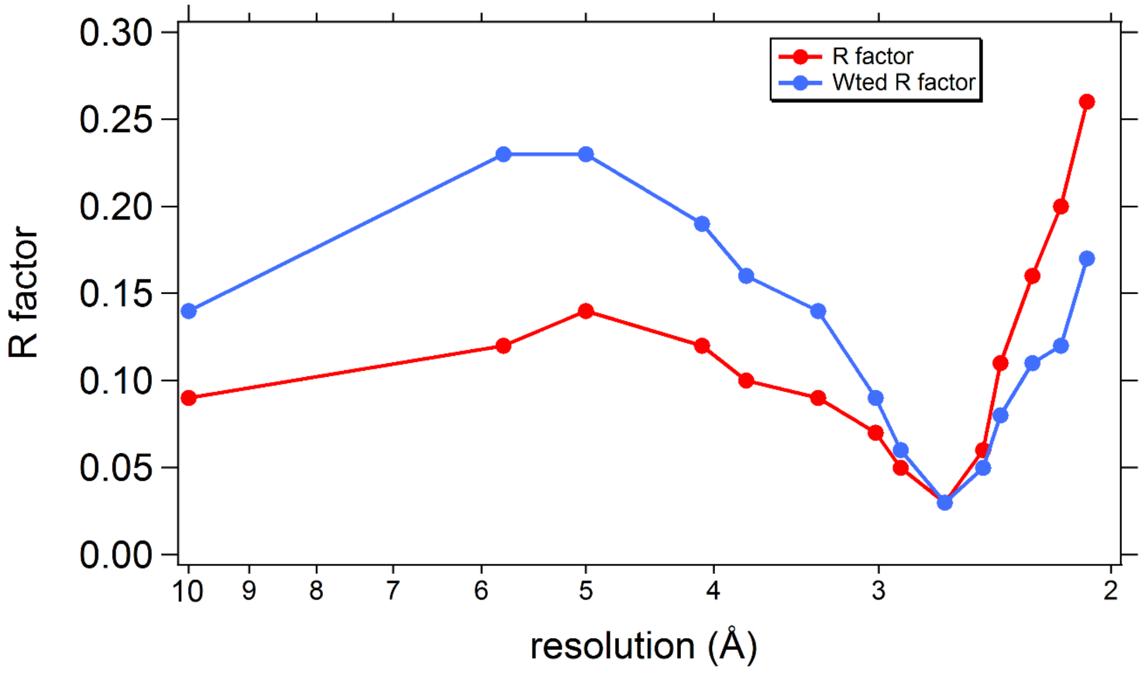

Figure S2 Phased difference Fourier map Fo(LF)-Fo(HF), superposed to the Gd-lysozyme model (Gd ions not shown) showing the ionization difference localized around the heavy atoms. Data to 2.1 Å, contoured at 4.5σ.

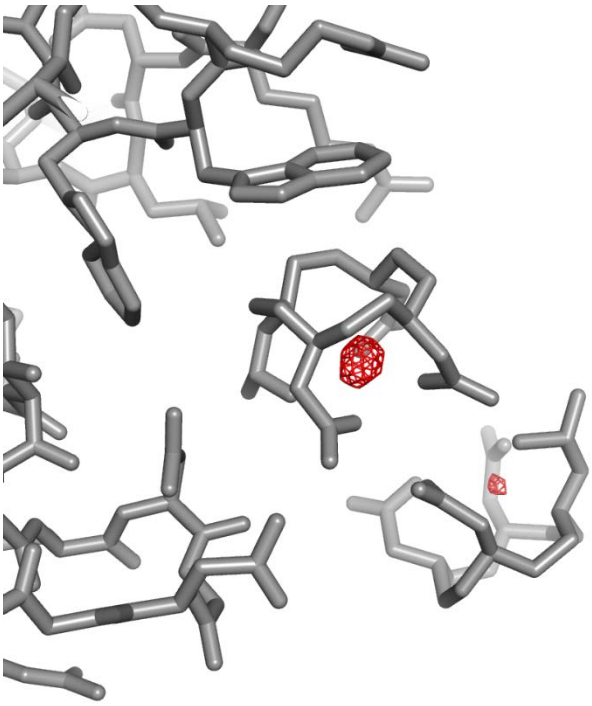

Figure S3 Average ionization of Fe atoms at the end of a 40 fs x-ray pulse, as a function of the x-ray fluence, simulated with a plasma physics code. The dotted line shows the average ionization of the atomic species in absence of secondary collisional ionization processes.

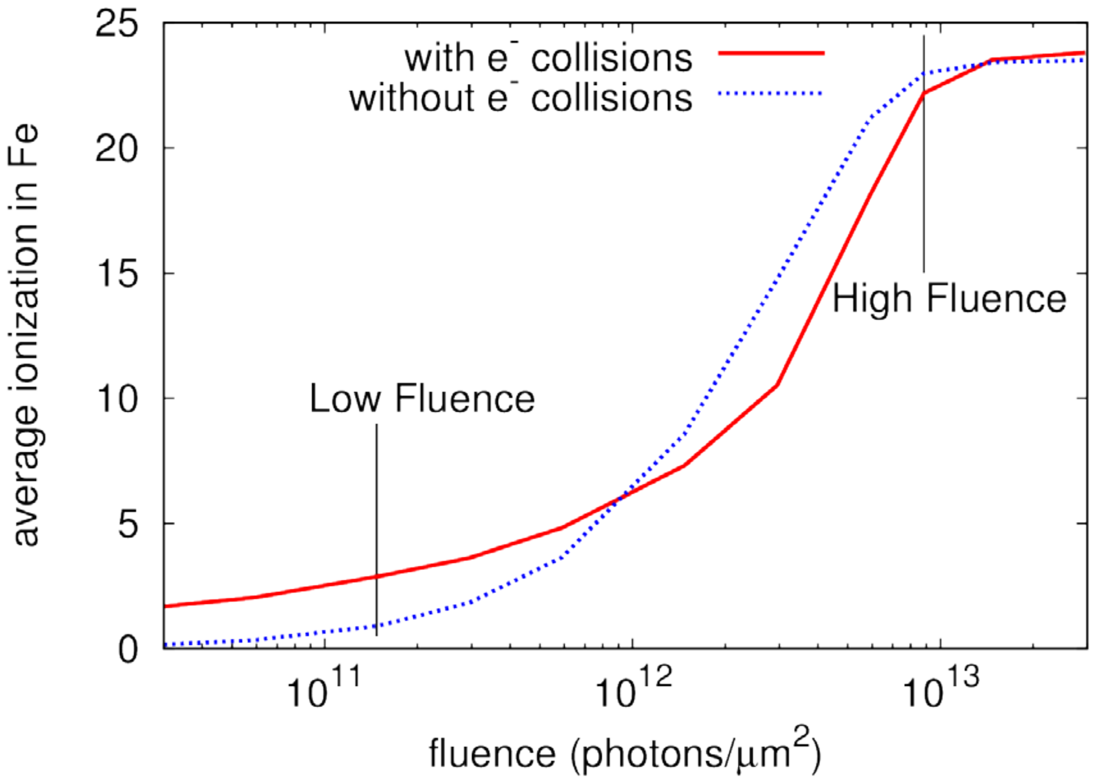

Supplement: Supplementary file 1 [file m-02-00627-sup1.pdf]
